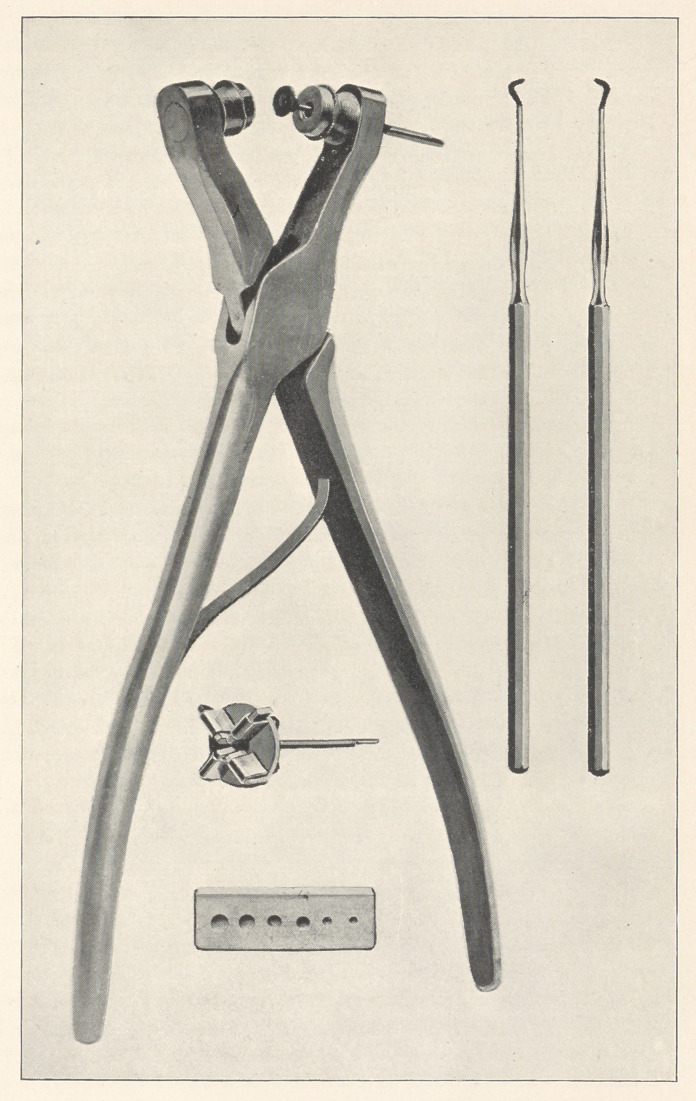# Some Things I Have Found Useful in Practice

**Published:** 1902-07

**Authors:** A. H. Stoddard

**Affiliations:** Boston, Mass.


### International Dental Journal.

Vol. XXIII. July, 1902. No. 7.

### Original Communications.[1]

### SOME THINGS I HAVE FOUND USEFUL IN PRACTICE.[2]

BY DR. A. H. STODDARD, BOSTON, MASS.

Mr. President and Gentlemen,—I have jotted down a few thoughts on some of the things that I have found of benefit in practice. Every man, after he has been in practice a few years, gathers certain instruments and methods which seem best suited to his needs, and become a part of his individuality. We are continually adding to and subtracting from this stock as the years go on. I have become very much attached to certain instruments which seemed indispensable, but when they were lost or broken I found others which replaced them. So with these methods and instruments I am to speak of, I may find others to take their place; but in the past, and at present, I am using them with more or less success.

Some few years ago Dr. Cooke recommended these right and left burnishers, which I will pass around, made by Mr. Grafrath, for

[1] The editor and publishers are not responsible for the views of authors of papers published in this department, nor for any claim to novelty, or otherwise, that may be made by them. No papers will be received for this department that have appeared in any other journal published in the country.

[2] Read before the Harvard Odontological Society, December 26, 1901.

plastic fillings in approximal spaces, particularly between molars and bicuspids. I have adopted them, and could not well dispense with them. You all may have used them, but if you have not, I should advise you to give them a trial.

A method of lining vulcanized plates with soft or velum rubber I have used for some time. I thought it was original with myself, but a short time ago I saw it described in one of the dental quarterlies. I can best describe its usefulness by passing around a sample which was made by the Boston Dental Laboratory for me, and by citing a few cases.

The first case, Mr. G., came to me a couple of years ago. He had full upper and lower dentures, the lower arch being very flat, the ridge having been absorbed almost entirely. The tissues were very sensitive, and it was impossible for him to eat with any comfort whatever. I made him an upper case first, with which he had no difficulty, and then I made a lower case and lined it with this soft rubber. I had to experiment somewhat in regard to the thickness, and finally I made it about four thicknesses before I had finished, and that gave quite a springy base. He was able to use it with great satisfaction, and has used it ever since. He broke the plate a short time ago, so I had occasion to see it again, and the rubber was in quite as good condition as it was when I first made it. That was about two years ago. He has taken good care of it; it was clean, and there was no disagreeable odor. Apparently it has been quite a success.

*Dr. Cooke.*—How many thicknesses in this plate?

*Dr. Stoddard.*—There were about four thicknesses of the velum rubber.

Another case, Mrs. K., came to me. She was unable to eat hard food at all, and was living on soups and soft foods. Her system was in very bad condition on account of it, and her physician told me that something must be done in the matter. She had had several lower plates, and she had a full upper denture as well. It was only the lower that gave trouble. The arch was a little better marked in that case, but the tissues were very sensitive indeed. After experimenting some I lined that case with four or five thicknesses of velum rubber, and she has been very comfortable ever since, so far as I know.

Another case, Mr. D., had lost all of his teeth back of the cuspids. He had been wearing a gold plate, fastened with clasps, but as he was unable to keep it up any longer, as the teeth that were clasped were lost, he had to resort to a suction plate. First, I made a gold suction plate, and he was not able to use it with any comfort; it kept dropping, etc. I finally recommended him to have a rubber plate, thinking I would get better suction with rubber, but he was not able to use that with any great success. I then made another rubber plate, and lined it with two thicknesses of velum rubber, I think, and he has been able to use that with much better success than any other plates he has had.

I had another case, Mrs. B., who was unable to keep up her plate by suction. It was rather a desperate case. She was quite an elderly lady, and it was necessary for her to wear her teeth in order to masticate properly and enunciate. And so I advised her, as a last resort, to use gum tragacanth every day sifted on to the plate. She kept it up for awhile until she got tired of it, and I then adopted this method in her case, and it has proved quite a success. She has been able to use this plate better than any that she has ever had before.

These cases, I think, prove its usefulness.

I have had some difficulty in getting corundum stones small enough to grind down gold fillings in fissure cavities in molars and bicuspids, and when I have set them on the mandrel with shellac they almost always come off before they are worn out. Therefore, for several years I have been in the habit of making a dozen or so corundum stones with this instrument which I will pass around, and you will notice the size and thinness of the stone. I have never had any of these come off in use, although I grind them clear down to the smallest point. Here are a couple worn down, as they are generally used. Sometimes, in finishing a gold filling, I use up a stone entirely.

I should have mentioned how I set them. The bits of corundum stone are warmed in a flame, and pressed in this manner, and an ordinary bur which has been worn out and become dull is used to set it on; then I squeeze it together like that, and the surplus corundum escapes around the sides. After it has cooled I put it in the engine and run it on a file for a moment to give me a sharp, clear, well-defined edge, and then I wash it in alcohol. You can do this only with corundum which is mixed with shellac.

Some time ago oxyphosphate of copper came out as a filling-material, but, having had experience with copper and amalgam, I

allowed others to do the experimenting. Dr. Payne and others told me that they had found this useful in saucer-shaped cavities, in buccal cavities, and others, where it was not advisable to use oxyphosphate of zinc, because it was liable to dissolve. My success with this method I can best describe by citing one or two cases. I will pass around this sample of oxyphosphate of copper.

In the first case Mr. C. had large cavities in the buccal surfaces of the third molars. It was impossible to fill them with gold, and the oxyphosphate of zinc cement washed out very readily, so that at the end of six months it was necessary to refill them. Dr. Payne suggested that I try the oxyphosphate of copper. It is extremely black, and very hard to manipulate. I used it in this case, perhaps a year ago. I have seen the same patient once or twice since, and I cannot see that there are any signs of dissolving, and the filling has been entirely satisfactory.

Another case, Mr. S., came to me a year ago in about the same condition, and I filled the teeth with this oxyphosphate of copper; and the last time I saw him they were in very good order.

It is very difficult to manipulate. It stains everything it touches. I mix it about as thick as thick cream. If it is much stiffer than that, it is impossible to use it satisfactorily; it becomes crumbly, but if about the thickness of thick cream it is very viscid. I carry it into the cavity on an old instrument, and then tamp it down with a piece of cotton which is moistened with saliva. If you use anything else, you will pull it out of the cavity as fast as you touch it; but in this way you can tamp it down and let it harden, and it makes a very desirable filling. But of course it is not to be used in any place except the back of the mouth, on account of its color.

A method I have used for making round inlays was shown at the Academy at the last meeting, and I will show it here for the benefit of those who did not see it at that time.

The instruments will explain themselves to a great extent. Here are a series of inlay burs, which are supplied by the dental supply companies. I have six sizes. Then I have taken a piece of ivory and cut six holes, to act as a gauge, which I keep to fit the inlays to, so that all the fitting can be done outside the mouth, and the filling can be carried into the mouth all complete. This appliance is made adjustable with carborundum pieces, made so that they can be set up to grind the porcelain rod to the desired size.

In making these inlays I first cut out the cavity in the tooth with an inverted cone or round bur. I select a size slightly smaller than one of these sizes which I have here on the gauge. Then I take a bur corresponding to the size in the gauge, and ream it out. The object in doing that is that if the cavity is cut out with the inlay bur, as soon as it strikes the bottom of the cavity it will move latterly and make the cavity elliptically, but if you use the inlay bur simply as a reamer, you get a perfectly round hole, and the inlay does not touch the bottom of the cavity at all.

Then I take a porcelain rod and grind it roughly on the lathe to correspond to the hole of the proper number in the gauge, except making it just a little bit larger, so it will not quite go into this hole in the gauge. I then set these points up, put it in the engine, and grind it down so it fits accurately into the gauge. After I have fitted it to the gauge, I use one of these thin carborundum disks and cut around the margin, snip it off, set it with cement, and polish it the same as any round inlay.

I will pass around this instrument and the gauge, and you will see I have made two or three inlays on one side. One was set with oxyphosphate of zinc, another one with Canada balsam, and two others are not set at all, but just turned in. This one which projects from the cavity is not set with anything, and you can see how tightly it clings to the margin of the cavity. This rod, which I will pass around, you will see fits into the hole in the gauge which I have marked.

The best rods for this purpose I think are made by Ash, of the English body. They are a better color and take a better polish when set. I will pass this around for your inspection.

## Figures and Tables

**Figure f1:**